# Qingda granule prevents Ang II-induced cardiac hypertrophy via inhibiting NF-κB signaling pathway

**DOI:** 10.3389/fphar.2025.1603316

**Published:** 2025-09-16

**Authors:** Yuhang Gong, Da Wo, Canran Wang, Ruokun Huang, En Ma, Celiang Wu, Jun Peng, Weidong Zhu, Dan-ni Ren

**Affiliations:** College of Integrative Medicine, Academy of Integrative Medicine, Fujian Key Laboratory of Integrative Medicine on Geriatric, Fujian University of Traditional Chinese Medicine, Fuzhou, Fujian, China

**Keywords:** Qingda granule, angiotensin II, hypertrophy, cardiac impairment, NF-κB signaling

## Abstract

**Background:**

Angiotensin II (Ang II) type 1 receptor (AT1R) signaling pathway is a key component of the renin-angiotensin-aldosterone system (RAAS) that is involved in the development of hypertension. Chronic Ang II overactivation results in pathological cardiac hypertrophy that progresses into decompensated cardiac dysfunction and impairment. Qingda granule (QDG) is a Traditional Chinese formula that has been used clinically in treating hypertension and its complications.

**Purpose:**

This study aimed to elucidate the role and underlying mechanisms of QDG in preventing Ang II-induced cardiac hypertrophy.

**Methods:**

We used chronic Ang II infusion via minipumps in mice and administered QDG daily to examine the effects of QDG on preventing hypertension and various parameters of cardiac impairment.

**Results:**

QDG treatment significantly reduced Ang II-induced elevation in blood pressure. Furthermore, QDG exerted a robust cardioprotective effect on chronic Ang II-induced cardiac hypertrophy and decompensated cardiac dysfunction. QDG also inhibited Ang II-induced adverse NF-κB signaling activation and downstream pro-inflammatory targets, which were prevented via administration with SC75741, a specific NF-κB inhibitor.

**Conclusion:**

Our findings provide further insight into the robust ability of QDG in preventing Ang II-induced cardiac hypertrophy via preventing NF-κB signaling activation and implicate its use in the clinical treatment of hypertension and cardiac hypertrophy.

## 1 Introduction

Cardiac hypertrophy primarily develops as an adaptive response to physiological and pathological stimuli (Nakamura and Sadoshima, 2018). However pathological hypertrophy can develop as a result of various chronic cardiovascular diseases such as hypertension, which results in decompensated cardiac function and ultimately resulting in heart failure (McMaster et al., 2015). The renin-angiotensin-aldosterone system (RAAS) is a key pathway in regulating blood pressure and maintaining homeostasis (Pugliese et al., 2020). Angiotensin II (Ang II) is a key member of RAAS, which plays a critical role in the development of hypertension and cardiac hypertrophy via binding to angiotensin II type 1 receptor (AT1R) (Bhullar and Dhalla, 2022). Studies have shown that Ang II not only promotes vasoconstriction but also contributes to cardiac fibrosis, inflammation, and apoptosis (García-Martín et al., 2021; Gu et al., 2021). Conversely, reduction of Ang II/AT1R expression prevents cardiac hypertrophy (Wei et al., 2023). Therefore, inhibiting Ang II/AT1R signaling pathway is a promising strategy for preventing hypertension-induced cardiac hypertrophy and its associated complications.

The NF-κB signaling pathway is mainly considered as the central mediator of the inflammatory process, but it has also been shown to be implicated in the development of pathological cardiac hypertrophy (Freund et al., 2005; Zelarayan et al., 2009; Nakamura and Sadoshima, 2018). NF-κB is a transcription factor composed of two subunits (P65 and P50), which is normally inactivated in the cytoplasm via binding to the inhibitory protein IκB, forming a trimeric complex. Studies indicate that Ang II induces the nuclear translocation of P65/P50 complexes via AT1R-mediated degradation of IκB, leading to disassembly of the trimer (Ruiz-Ortega et al., 2000). Additionally, overexpression of AT1R has been shown to promote P65 nuclear translocation in mice. Given the pivotal role of the NF-κB signaling pathway in pathological cardiac hypertrophy, identifying novel drugs that can not only inhibit Ang II/AT1R signaling but also prevent adverse effects associated with NF-κB signaling activation may be critical.

Qingda granule (QDG) is a Traditional Chinese Medicine formula that has been used in clinical practice for treating hypertension and its associated cardiac complications. QDG is composed of *Gastrodia elata* Blume (Tianma), *Uncaria rhynchophylla* (Miz.) Miz. ex Havil. (Gouteng), *Scutelaria baicalensis* Georgi (Huangqin), and *Nelumbo nucifera* Gaertn. (Lianzixin) and has been shown to be effective in preventing hypertension, alleviating cardiac hypertrophy (Gao et al., 2022) and reducing inflammation (Chen et al., 2022). However, the mechanisms by which QDG attenuates cardiac hypertrophy remain largely unelucidated. This study aimed to clarify the mechanisms underlying the protective effects of QDG in cardiac hypertrophy, in particular, its effects in preventing the adverse activation of NF-κB signaling pathway.

## 2 Methods

### 2.1 QDG preparation

QDG was provided by Jiangyin Tianjiang Pharmaceutical Co., Ltd. (Jiangsu, China; Batch No. 2012334). QDG was prepared as the combination of four botanical drugs (*Gastrodia elata* Blume (Tianma): *Uncaria rhynchophylla* (Miz.) Miz. ex Havil. (Gouteng): *Scutelaria baicalensis* Georgi (Huangqin): *Nelumbo nucifera* Gaertn. (Lianzixin) at a ratio of 12:10:6:5. QDG was dissolved in phosphate buffered saline (PBS, Wisent Corporation, Canada) solution via ultrasonication and administered at two different concentrations: 0.9 g/kg/day and 1.8 g/kg/day, based on the dose conversion formula for animals and humans (Wojcikowski and Gobe, 2014). For animal studies, QDG or an equivalent dosage of PBS were administered via oral gavage, daily. For *in vitro* experiments, QDG was used at the following concentrations: 0.05 mg/mL and 0.1 mg/mL.

### 2.2 High-performance liquid chromatography-tandem mass spectrometry (HPLC-MS/MS)

Chemical fingerprint analysis of QDG extract was conducted using HPLC-MS/MS, to determine the chemical profile of top bioactive metabolites in QDG. Briefly, 0.3 g QDG powder extract was prepared at a concentration of (1 mg/mL), filtered (0.22 μm) and injected into the HPLC-MS/MS system along with the respective standards (LC-30A, Japan). Subsequently, gradient elution procedure was used to separate these solutions on a C18 ODS column (1.8 μm, 2.1 × 100 mm) with 0.2% 2-sulfobenzoic acid hydrate (A) and acetonitrile (B) as mobile phase. The gradient program was: 0 min (97:5), 0.01 min (75:30), 37 min (95:5), at 0.5 mL/min flow rate.

### 2.3 Animals model

All animal experiments in this study were approved by the Institutional Animal Care and Use Committee of Fujian University of Traditional Chinese Medicine and conducted in accordance with the Guide for the Care and Use of Laboratory Animals from the National Institutes of Health and ARRIVE (Animal Research: Reporting of *In Vivo* Experiments) guidelines. 8-week-old C57BL/6 male mice were purchased from SLAC Laboratory Animal Technology Co. Ltd. (Shanghai, China) and kept in SPF3 grade center of Fujian University of Fujian Traditional Chinese Medicine. Prior to model, mice had an average weight of 25 g, had visibly healthy appearance and normal cardiac function via baseline echocardiography assessment.

PBS or Ang II (1,000 ng/kg/min) (MCE, United States) was administered via Alzet osmotic minipumps (Model 2004) purchased from Alza Corporation (Palo Alto, Ca, United States) and used in accordance with the manufacturer’s instructions, at the dosage previously reported for modeling Ang II-induced cardiac hypertrophy (Wang et al., 2023; Ye et al., 2025). Mice were randomized into four groups: (1) Control; (2) Ang II + PBS; (3) Ang II + QDG (0.9 g/kg/day); (4) Ang II + QDG (1.8 g/kg/day). Sample size was 14 mice/group and subsequent experiments were performed in parallel, with eight mice/group allocated for physiological and histological assessments and 6 mice/group allocated to investigation of molecular mechanisms. QDG treatments were administered daily via oral gavage. Control groups were administered PBS via oral gavage. For Ang II model, osmotic minipumps were implanted into the back of mice, which were filled with PBS for control group and Ang II for the model groups. A separate short-timepoint animal model used the same grouping with 6 mice/group, where QDG was administered for 3 days, followed by intraperitoneal injection of Ang II and subsequent collection of tissue samples at 10 min post-injection.

### 2.4 Blood pressure measurement

Mouse blood pressures were measured via non-invasive tail-vein blood pressure instrument (Kent Scientific, United States) according to the manufacturer’s instructions. Results were obtained from the mean of at least 10 valid cuff inflation-deflation cycles with distinct sigmoidal systolic measurement curves.

### 2.5 Echocardiography

Echocardiography was performed using Visual Sonics Vevo 2100 Imaging System. Mice were anaesthetized via inhaled isoflurane (Sigma Aldrich, United States) using a vaporizer (EZ Anesthesia). M-mode measurements taken from parasternal long axis view were used to determine left ventricular (LV) dimensions, including left ventricular posterior wall in diastole (LVPW; d) and interventricular septum at end diastole (IVS; d). LV ejection fraction (EF%) and fractional shortening (FS%) were derived from Vevo Lab.

### 2.6 Cell culture and treatments

Neonatal rat cardiomyocytes (NRCMs) were derived from 1- to 2-day-old rats according to standard protocol. Briefly, hearts were excised and digested with 0.1% trypsin (Gibco, United States) in a 37 °C magnetic stirrer bath (Thermo Fisher, United States). The cardiomyocyte H9c2 cell line was also used as a separate cardiomyocyte cell line to support the results from primary neonatal cardiomyocytes. HEK293-AT1R renal cell line that overexpresses the AT1R receptor was chosen to study the effect of QDG in the presence of AT1R overactivation *in vitro*. All cells were cultured in a 37 °C incubator supplemented with 5% carbon dioxide. For H9c2 and HEK293-AT1R cell lines, third-passage cells were used for experimental grouping (see Results for details). All Ang II-treated groups received identical stimulation with 1 μM Ang II, and TNF-α treatment groups were administered with 10 ng/mL TNF-α. 10 μM SC75741 was used within relevant groups.

### 2.7 CCK-8 assay

NRCMs were seeded in 96-well plates and treated with different QDG concentrations for 24 h. Cell culture medium was replaced with a 1:10 dilution of CCK-8 (APExBio, United States) solution in fresh medium. Following 1–3 h of incubation in the dark, the resulting absorbances were measured at 450 nm and 620 nm by using Microplate Reader (Thermo Fisher, United States) and subsequently, cell viability was determined.

### 2.8 Histology

Hearts were submerged in 4% paraformaldehyde (PFA) (Biosharp, China) for 24 h, then dehydrated, cleared, and embedded in paraffin. Left ventricles of mouse hearts were used for histological sectioning and cut into 5 μm sections. The degree of collagen deposition was detected by Masson’s trichrome staining (Aladdin scientific, China) and myocyte cross-sectional areas were detected by Wheat germ agglutinin (WGA) (Thermo Fisher, United States) staining according to standard protocol. Sections were observed under optical or fluorescence microscope with ×20 objective lens.

### 2.9 Quantitative real-time polymerase chain reaction (QPCR)

QPCR was used to detect the mRNA expression levels of atrial natriuretic peptide (ANP), brain natriuretic peptide (BNP), alpha-skeletal actin (ACTA1), *IL-1*β, *TNF-*α, collagen I and collagen III in mouse hearts, as well as the mRNA expression levels of ANP and BNP mRNA expression level in cardiomyocytes. GAPDH was used as the reference gene for determination of relative gene expressions. The primer sequences were as follows:

Mouse ANP forward 5′-GGA​GCC​TAC​GAA​GAT​CCA​GC-3’; mouse ANP reverse 5′-TCC​AAT​CCT​GTC​AAT​CCT​ACC​C-3’.

Mouse BNP forward 5′-CTT​CGG​TCT​CAA​GGC​AGC​AC-3’; mouse BNP reverse 5′-GCCCAAACGACTGACG GATC-3’.

Mouse ACTA1 forward 5′-ATG​GAT​TCC​CGT​TCG​AGT​AC-3’; mouse ACTA1 reverse 5′-TCAGCTGGATAGCGAC ATCG-3’

Mouse *IL-1*β forward 5′-GAA​ATG​CCA​CCT​TTT​GAC​AGT​G-3’; mouse *IL-1*β reverse 5′-TGG​ATG​CTC​TCA​TCA​GGA​CAG-3’.

Mouse *TNF-*α forward 5′-TGT​AGC​CCA​CGT​CGT​AGC​AAA-3’; mouse *TNF-*α reverse 5′-CTG​GCA​CCA​CTA​GTT​GGT​TGT-3’

Mouse collagen I forward 5′-ATG​GAT​TCC​CGT​TCG​AGT​AC-3’; mouse collagen I reverse 5′-TCAGCTGGATAGCGAC ATCG-3’.

Mouse collagen III forward 5′-CGT​AGA​TGA​ATT​GGG​ATG​CA-3’; mouse collagen III reverse 5′-ACA​TGG​TTC​TGG​CTT​CCA​G-3’

Mouse GAPDH forward 5′-TGG​CCT​TCC​GTG​TTC​CTA​C-3’; mouse GAPDH reverse 5’ -GAGTTGCTGTTGAAGT CGCA-3’.

Rat ANP forward 5′-CCA​AGG​GCT​TCT​TCC​TCT​TCC-3’; rat ANP reverse 5′-TCT​TCT​CCT​CCA​GGT​GGT​CTA​G-3’.

Rat BNP forward 5′-CTG​GGA​AGT​CCT​AGC​CAG​TCT​CCA-3’; rat BNP reverse 5′-GCG​ACT​GAC​TGC​GCC​GAT​CCG​GTC-3’.

Rat GAPDH forward 5′-CCA​TCA​ACG​ACC​CCT​TCA​TT-3’; rat GAPDH reverse 5′-GAC​CAG​CTT​CCC​ATT​CTC​AG-3’.

### 2.10 Enzyme linked immunosorbent assay (ELISA)

Level of blood serum BNP was determined using BNP ELISA kit (Enzyme-linked Biotechnology Co., Ltd., China, Cat. no. ml037594) according to the manufacturer’s instructions. Briefly, standards and serum samples were diluted with sample diluent at the recommended dilution factors, incubated with the respective enzyme-labeled antibodies at 37 °C, and the resulting optical densities were determined at 450 nm using a Microplate Reader. Final serum concentrations of BNP were calculated as picograms per milliliter.

### 2.11 Western blot

Proteins were extracted using total extraction kit (Beyotime, China) as well as nucleoprotein extraction kit (Sangon Biotech, China; Cat. no. C500009). Concentration of all proteins were detected by bicinchoninic acid (BCA) assay prior to Western blot. Briefly, proteins were separated via Sodium Dodecyl Sulfate Polyacrylamide Gel Electrophoresis (SDS-PAGE), transferred to 0.22 µm PVDF membrane and blocked with 5% non-fat milk and incubated with the following primary antibodies overnight at 4 °C: rabbit monoclonal anti-TATA binding protein (TBP) (1:1,000, CST, United States; Cat. no. 44059S), anti-P65 (1:1,000, CST, United States; Cat. no. 8242S), anti-phospho-P65 (1:1,000, CST, United States; Cat. no. 3033S), anti-IκB (1:1,000, CST, United States; Cat. no. 4812S), anti-phospho-IκB (1:800, CST, United States; Cat. no. 2859S), anti-β-actin (1:1,000, CST, United States; Cat. no. 4970), anti-extracellular signal-regulated kinases (ERK) 1/2 (1:1,000, CST, United States; Cat. No. 4695S), anti-phospho-ERK 1/2 (1:1,000, CST, United States; Cat. No. 4370S), and mouse monoclonal anti-GAPDH (1:5,000, CST, United States; Cat. No. 97166S). Subsequently, membranes were incubated with the respective HRP-conjugated secondary antibodies and bands were detected via chemiluminescence.

### 2.12 Immunofluorescence

NRCMs were seeded in immunofluorescent dish, then fixed with 4% PFA (Sigma, United States) for 15 min and permeabilized with 0.25% Triton X-100 (Sigma, United States), blocked in 5% BSA (Roche, United States) for 1 h and incubated overnight at 4 °C with the following primary antibody: rabbit monoclonal cardiac Troponin T (cTnT, 1:800, Sigma, United States; Cat. No. SAB5702554) and respective anti-rabbit secondary antibody conjugated to Alexa Fluor 647 (1:400, Abcam, United States; Cat. No. ab150115). Subsequently, samples were mounted in DAPI fluorescence mounting medium.

### 2.13 Statistical analysis

Statistical analyses were performed using GraphPad Prism 9.5.0. Shapiro-Wilk tests for normality were performed to ensure outcomes with normal distribution, and thereafter, comparisons of means were performed using one-way ANOVA followed by Tukey’s post-hoc analysis. Data are presented as mean ± standard error of the mean.

## 3 Results

### 3.1 Chemical fingerprint of QDG extract using HPLC

The chemical profiles of the top bioactive metabolites in QDG extract were determined by HPLC analysis and quantified using calibration curves of the corresponding chemical standards (Supplementary Figure S1). The top bioactive metabolites were gastrodin (7.87 mg/g dry QDG extract, main peak at 12.973 min) from *Gastrodia elata* Blume (Tianma), baicalin (50.13 mg/g dry QDG extract, main peak at 9.134 min) from *Scutelaria baicalensis* Georgi (Huangqin), rhynocholphylline (0.20 mg/g dry QDG extract, main peak at 5.638 min) from *Uncaria rhynchophylla* (Miz.) Miz. ex Havil. (Gouteng), and liensinine (88.75 mg/g dry QDG extract, main peak at (main peak at 3.026 min) from *Nelumbo nucifera* Gaertn. (Lianzixin).

### 3.2 QDG prevents Ang II-induced hypertension and hypertrophic response

**FIGURE 1:**
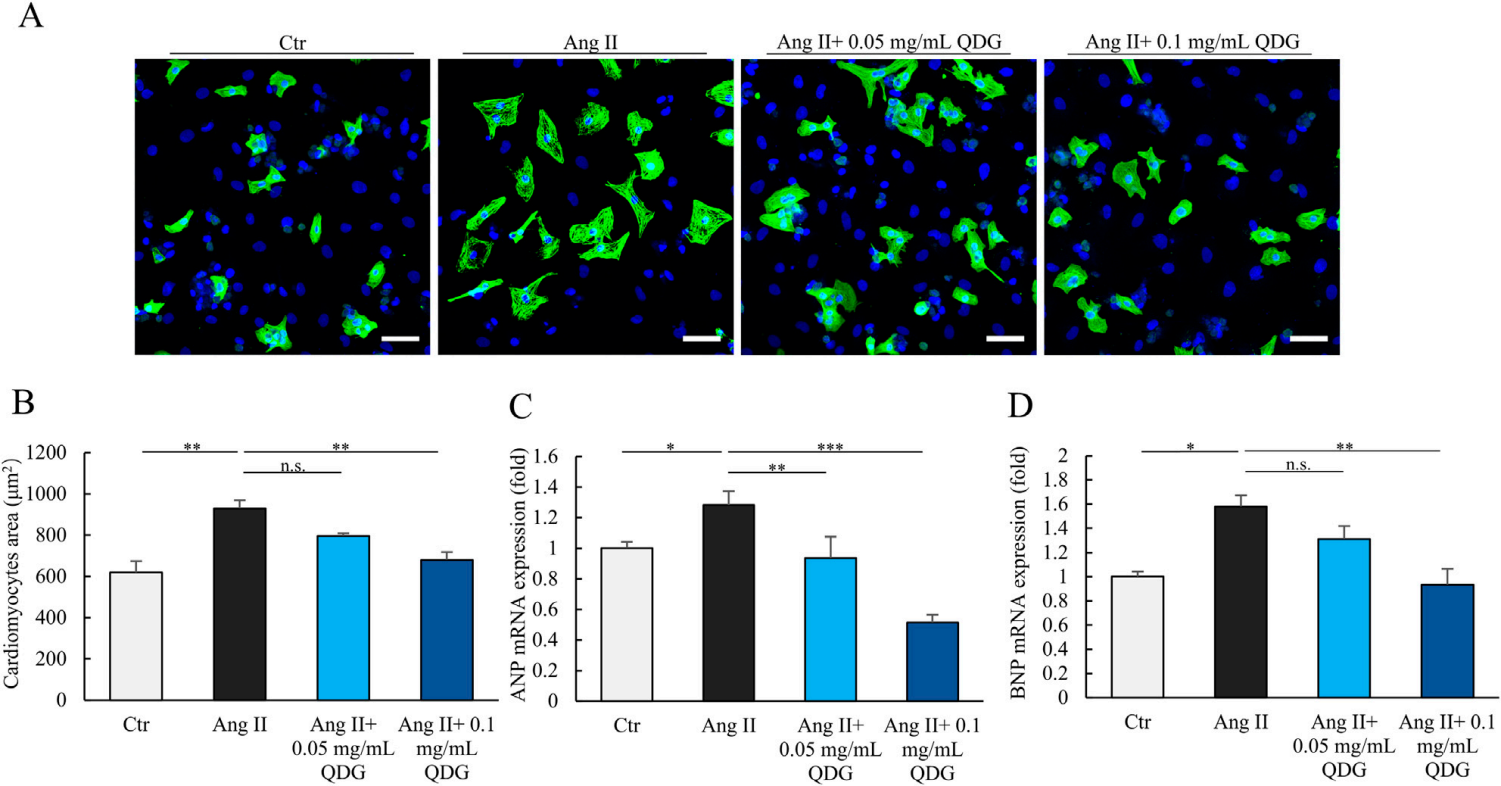
QDG prevents hypertrophic response in cardiomyocytes. **(A)** Immunofluorescence images of NRCMs pretreated with QDG (0.05 mg/mL, 0.1 mg/mL) for 24 h and then treated with Ang II (1 μM) for 24 h. Cardiac-Troponin T (cTnT, green) staining was performed to identify cells, DAPI (blue). Scale bar, 75 µm. **(B)** Quantification of cell area. NRCMs treated as in **(A)**. n = 3. n.s., no significance, **P < 0.01 vs. Ang II alone. **(C,D)** ANP **(C)** and BNP **(D)** mRNA levels in NRCMs. NRCMs treated as in **(A)**. n = 3. n.s., no significance *P < 0.05, **P < 0.01, ***P < 0.001 vs. Ang II alone.

We first investigated the potential impact of QDG on cardiac hypertrophy using cultured neonatal NRCMs treated with Ang II. Immunofluorescence analysis revealed that treatment with Ang II resulted in a significant increase in the size of NRCMs (Figure 1A). Interestingly, cardiomyocytes that were pre-treated with QDG for 24 h prior to Ang II stimulation, effectively attenuated the Ang II-induced increase in the size of NRCMs (Figures 1A,B). Furthermore, Ang II stimulation significantly increased the mRNA levels of cardiac hypertrophy markers, including ANP and BNP, which were markedly attenuated by QDG treatment (Figures 1C,D). These results indicate that QDG can effectively prevent Ang II-induced hypertrophic response in cardiomyocytes.

**FIGURE 2:**
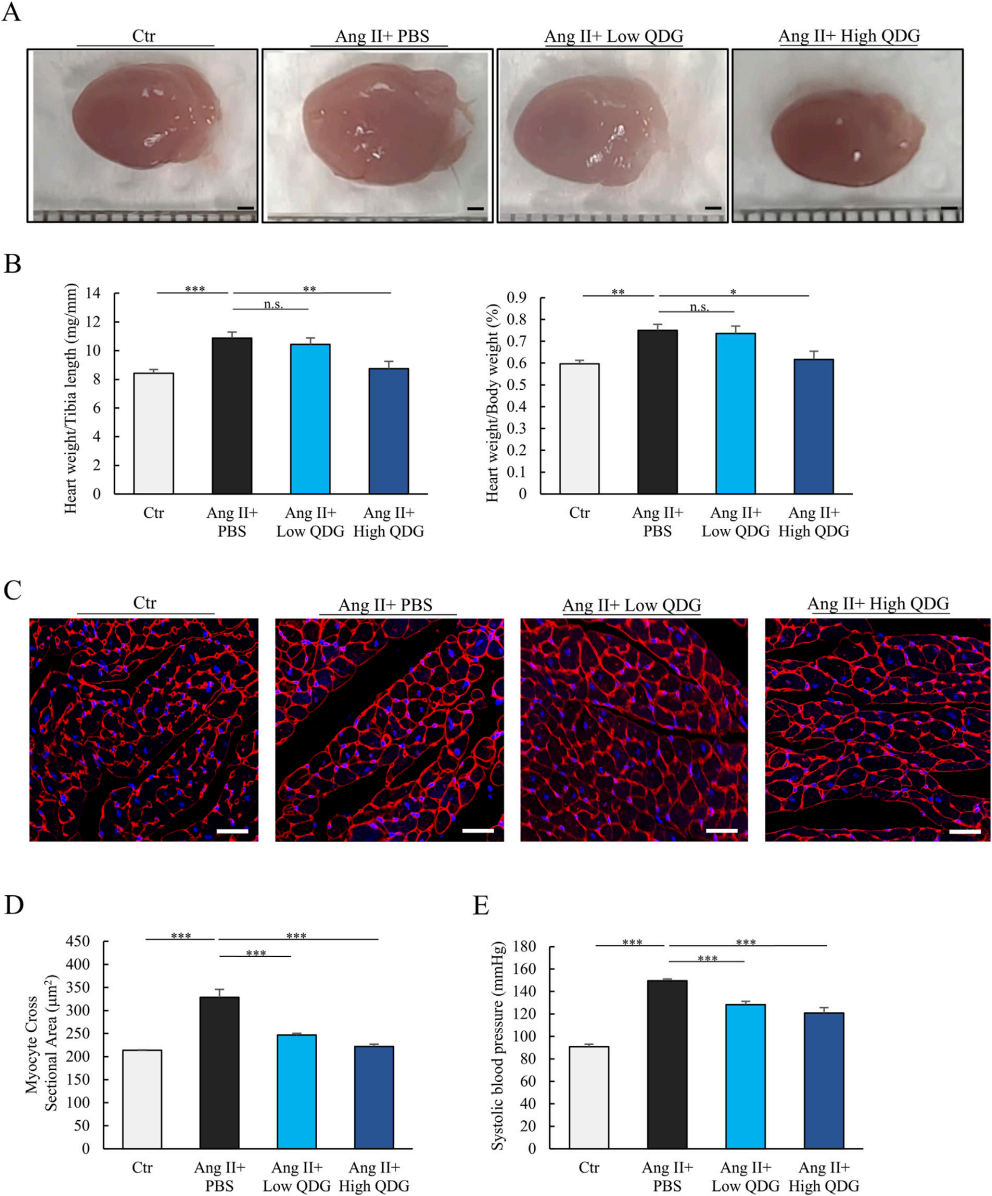
QDG prevents Ang II-induced hypertension and hypertrophic response in mice. **(A)** Heart size (scale bar, 1 mm) showing cardiac hypertrophy in mice at 4 weeks post-Ang II infusion in mice administrated with PBS or QDG. **(B)** Ratios of heart weight-to-tibia length (HW/TL) or heart weight-to-body weight (HW/BW) in mice treated in **(A)**. n = 8. n.s., no significance *P < 0.05, **P < 0.01, ***P < 0.001 vs. Ang II + PBS. **(C)** WGA staining (scale bar, 50 μm) showing cardiac hypertrophy in mice treated in **(A)**. **(D)** Quantification of myocyte cross sectional area in mice treated as in **(A)**. n = 8. ***P < 0.001 vs. Ang II + PBS. **(E)** Systolic blood pressure at 4 weeks post-Ang II infusion in mice administrated with PBS or QDG. n = 8. **P < 0.01, ***P < 0.001 vs. Ang II + PBS.

Next, we further examined the protective effects of QDG on Ang II-induced cardiac hypertrophy and hypertension in mice. Similar to the hypertrophic response in cardiomyocytes, Ang II-implanted mice showed significant enlargement of the heart, which was markedly attenuated by mice co-administered daily with QDG (Figure 2A). Both heart weight/tibia length and heart weight/body weight ratios also exhibited a similar trend (Figure 2B). WGA staining of the heart further demonstrated that Ang II stimulated cardiomyocyte hypertrophy, with significantly increased myocyte cross-sectional area, which was attenuated by QDG treatment (Figures 2C,D). Moreover, QDG treatment significantly decreased the Ang II-induced elevation in blood pressure after 4 weeks (Figure 2E). Taken together, these results demonstrate that QDG administration can effectively prevent Ang II-induced hypertension and cardiac hypertrophic response.

### 3.3 QDG inhibits Ang II-induced cardiac fibrosis and dysfunction

**FIGURE 3:**
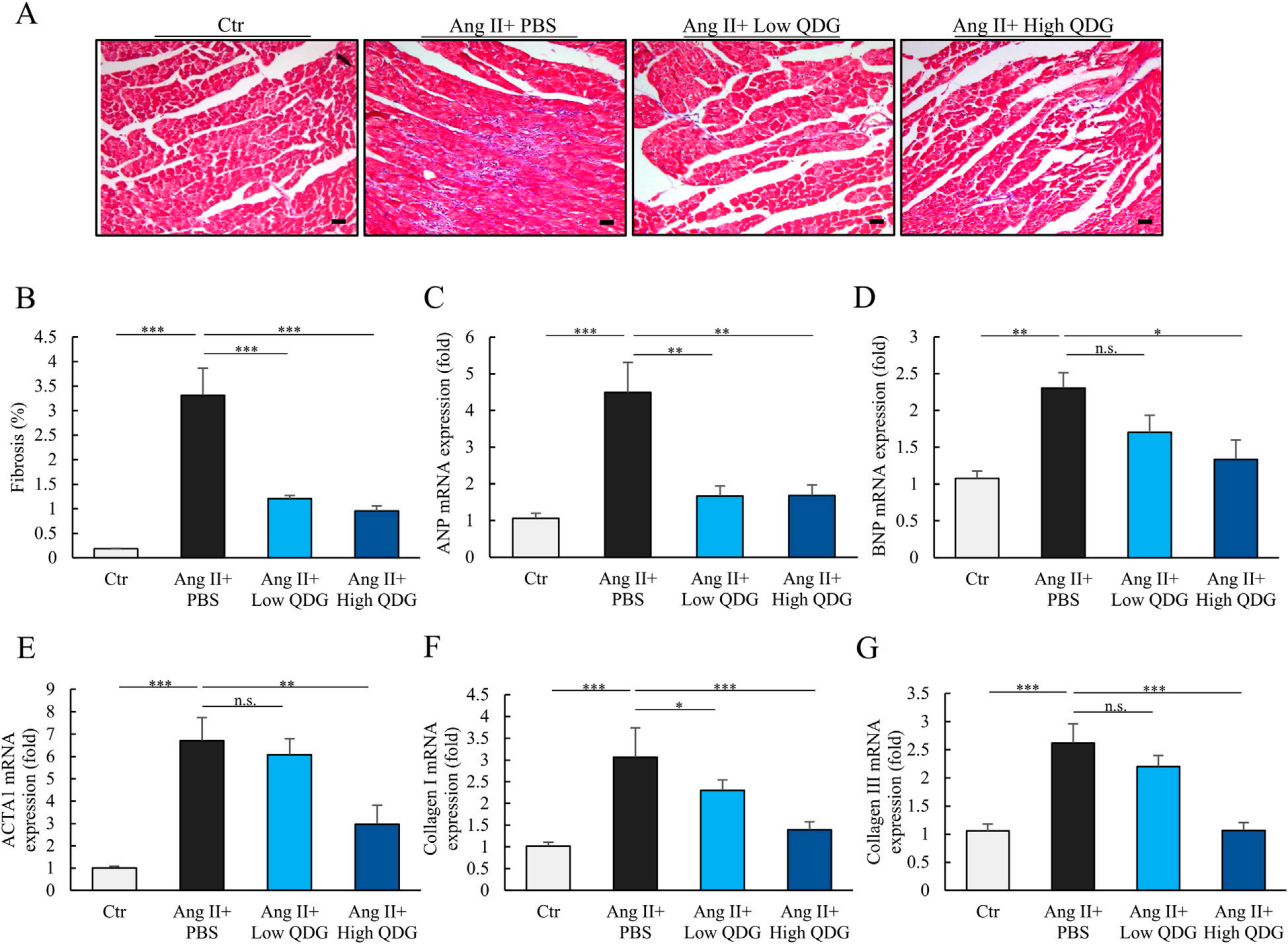
QDG inhibits Ang II-induced cardiac fibrosis and dysfunction. **(A)** Masson’s Trichrome Staining (scale bar, 50 µm) showing cardiac hypertrophy and cardiac fibrosis in mice administrated with PBS or QDG at 4 weeks post-Ang II infusion. **(B)** Quantification of cardiac fibrosis in mice treated as in **(A)**. n = 8. ***P < 0.001 vs. Ang II + PBS. **(C–E)** ANP **(C)**, BNP **(D)** and ACTA1 **(E)** mRNA levels in the heart of mice treated as in **(A)**. n = 6. n.s., no significance *P < 0.05, **P < 0.01, ***P < 0.001 vs. Ang II + PBS. **(F,G)** Collagen I and collagen III mRNA levels in the heart of mice treated as in **(A)**. n = 6. n.s., no significance *P < 0.05, ***P < 0.001 vs. Ang II + PBS.

Masson’s trichrome staining revealed extensive interstitial fibrosis in the hearts of mice treated with Ang II for 4 weeks (Figure 3A). In contrast, mice administered with QDG had significantly reduced degree of cardiac fibrosis (Figures 3A,B). Ang II-treated mice also showed significantly upregulated mRNA levels of cardiac dysfunction and hypertrophy markers, including ANP, BNP, and ACTA1 (Figures 3C–E). In addition, Ang II induction also induced significant mRNA upregulation of the major cardiac fibrosis markers, collagen I and collagen III (Figures 3F,G). Importantly, mice administered with QDG significantly attenuated the Ang II-induced mRNA elevations of these markers in the heart (Figures 3C–G). Moreover, serum BNP levels were significantly elevated at 4-week following Ang II induction, while mice that received QDG administration significantly attenuated the Ang II-induced increase in BNP in a dose-dependent manner (Supplementary Figure S2). These results further demonstrate that QDG can effectively prevent Ang II-induced cardiac dysfunction, hypertrophy, and cardiac fibrosis in mice.

A concurrent outcome of chronic Ang II induction is kidney damage, most notably hypertension-related renal injury and fibrosis. Masson’s trichrome staining showed that Ang II induction for 4 weeks resulted in significant areas of renal fibrosis, while mice administered with QDG markedly decreased the degree of fibrosis, in a dose-dependent manner (Supplementary Figure S3A,S3B). Moreover, biochemical analyses of renal injury markers serum creatinine and blood urea nitrogen (BUN) were significantly elevated following Ang II induction, which were markedly attenuated in mice administered with QDG, especially at a high dose (Supplementary Figure S3C,S3D). Taken together, these results demonstrated that QDG also exerted renal protective effects following chronic Ang II induction.

### 3.4 QDG prevents Ang II-induced cardiac impairment

**FIGURE 4:**
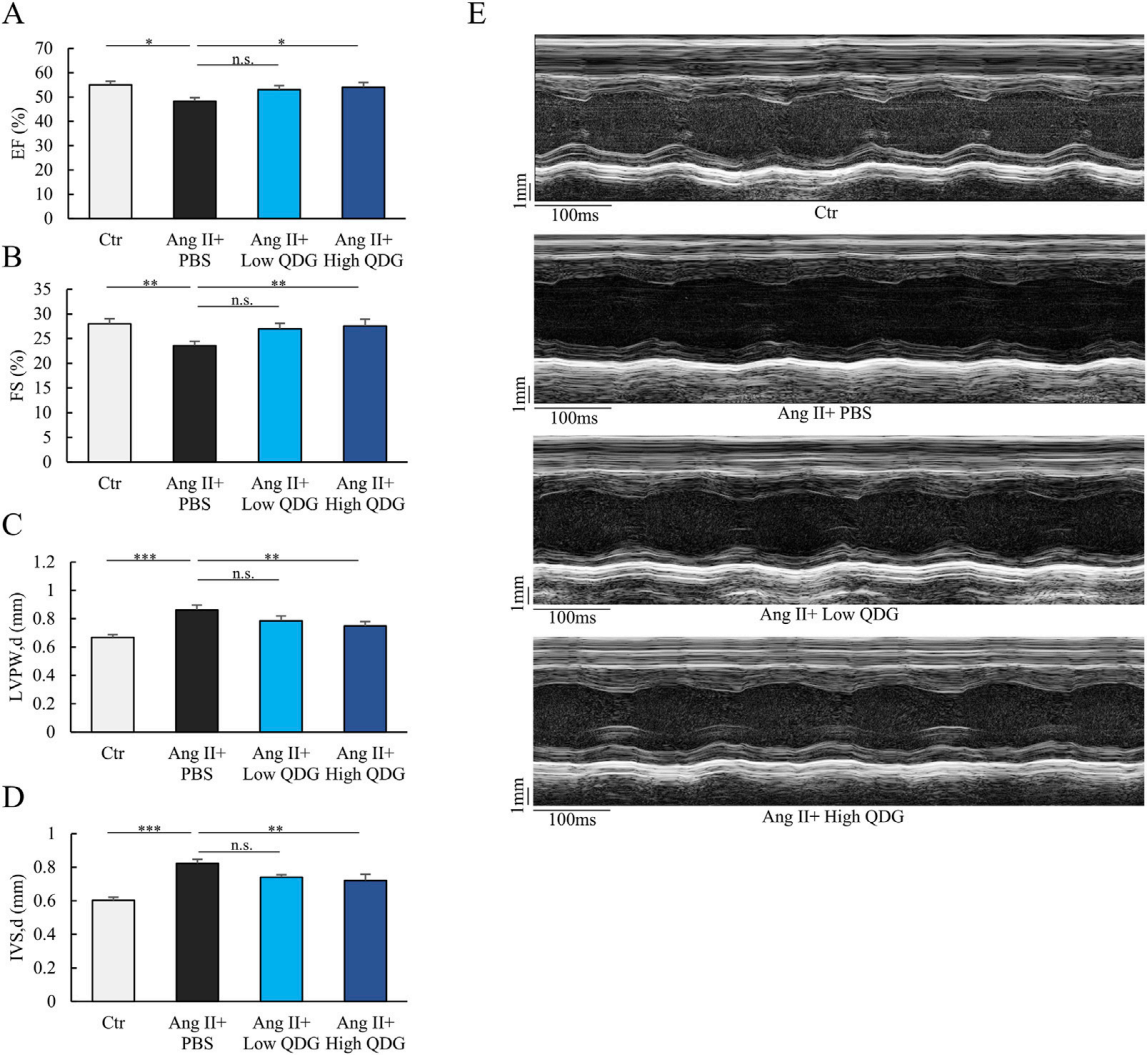
QDG prevents Ang II-induced cardiac impairment. **(A,B)** Echocardiographic assessment of ejection fraction **(A)** and fractional shortening **(B)** at 4 weeks post-Ang II infusion in mice administrated with PBS or QDG. n = 8. n.s., no significance *P < 0.05, **P < 0.01 vs. Ang II + PBS. **(C,D)** Left ventricular posterior wall end diastole (LVPW; d)** (C)** and intact ventricular septum end diastole (IVS; d) **(D)** in mice treated as in **(A,B)**. n = 8. n.s., no significance, **P < 0.01, ***P < 0.001 vs. Ang II + PBS. **(E)** M-mode echocardiography in mice treated as in **(A,B)**.

Echocardiography assessment showed that after 4 weeks of Ang II infusion, mice exhibited significant reductions in left ventricular (LV) ejection fraction (EF%) and fractional shortening (FS%) parameters (Figures 4A,B). Notably, mice treated with QDG had markedly improved cardiac function following Ang II induction for 4 weeks (Figures 4A,B). Chronic Ang II infusion for 4 weeks resulted in a marked increase in LV wall thickness, in both the posterior wall (LVPW; d, Figure 4C), as well as the interventricular septum at end-diastole (IVS; d, Figure 4D). QDG treatment effectively prevented these adverse structural changes in the heart, further supporting its ability to mitigate Ang II-induced cardiac hypertrophy (Figures 4C,D). M-mode evaluation also revealed impaired LV wall motion in Ang II-treated mice, which was alleviated in mice treated with QDG (Figure 4E). These findings further demonstrate that QDG administration can not only prevent chronic Ang II-induced cardiac hypertrophy, but also effectively protect against cardiac impairment in mice.

### 3.5 QDG attenuates Ang II/AT1R signaling transduction

**FIGURE 5:**
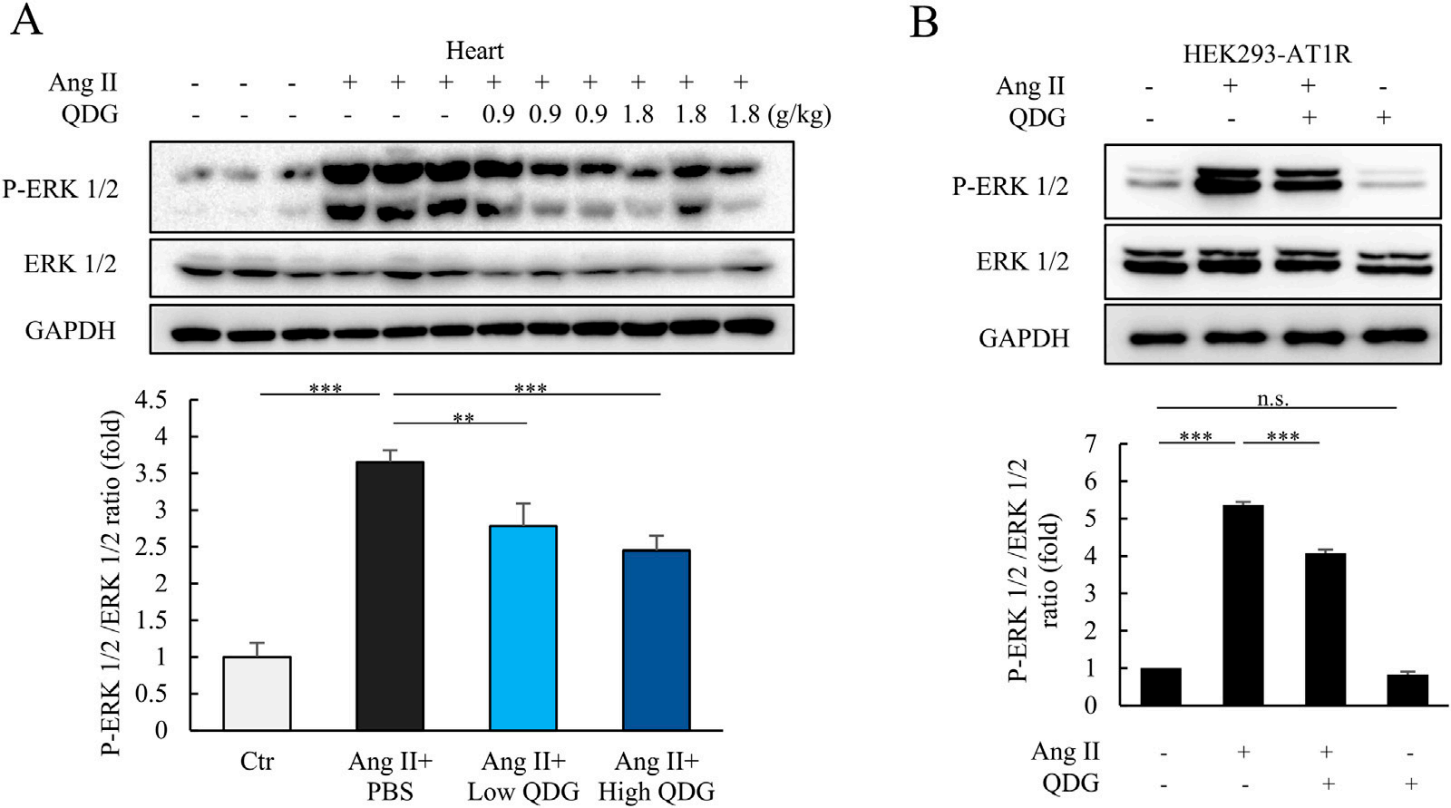
QDG attenuates Ang II/AT1R signaling transduction. **(A)** Representative immunoblots (upper) and quantification (lower) of P-ERK 1/2 expression in the heart of mice administrated with QDG (0.9 g/kg/day, 1.8 g/kg/day) or PBS for 3 days, and subsequent injection with Ang II (10 μg/kg, i.p.) for 10 min n = 6. n.s., no significance, **P < 0.01, ***P < 0.001 vs Ang II + PBS. **(B)** Representative immunoblots (upper) and quantification (lower) of P-ERK 1/2 expression in HEK293-AT1R pretreated with QDG (0.1 mg/mL) for 6 h then treated with Ang II (1 μM) for 8 min n = 3. ***P < 0.001 vs. Ang II alone.

We further investigated the effect of QDG on Ang II/AT1R signaling transduction during cardiac hypertrophy. Indeed, we found that Ang II administration caused rapid increases in the levels of phosphorylated ERK 1/2 (P-ERK 1/2) – the downstream targets of Ang II/AT1R signaling within 10 min in mice (Figure 5A). Notably, mice that were pre-administered with QDG for three consecutive days significantly attenuated the Ang II-induced phosphorylation of ERK 1/2 (Figure 5A). Furthermore, *in vitro* experiments using HEK293-AT1R cells showed that Ang II treatment induced robust phosphorylation of ERK 1/2 within 8 min, which was significantly decreased in cells pre-treated with QDG for 6 h (Figure 5B). These findings demonstrate that QDG attenuates Ang II/AT1R signaling transduction and downstream target activations both *in vivo* and *in vitro*.

### 3.6 QDG inhibits Ang II/AT1R induced NF-κB signaling activation

**FIGURE 6:**
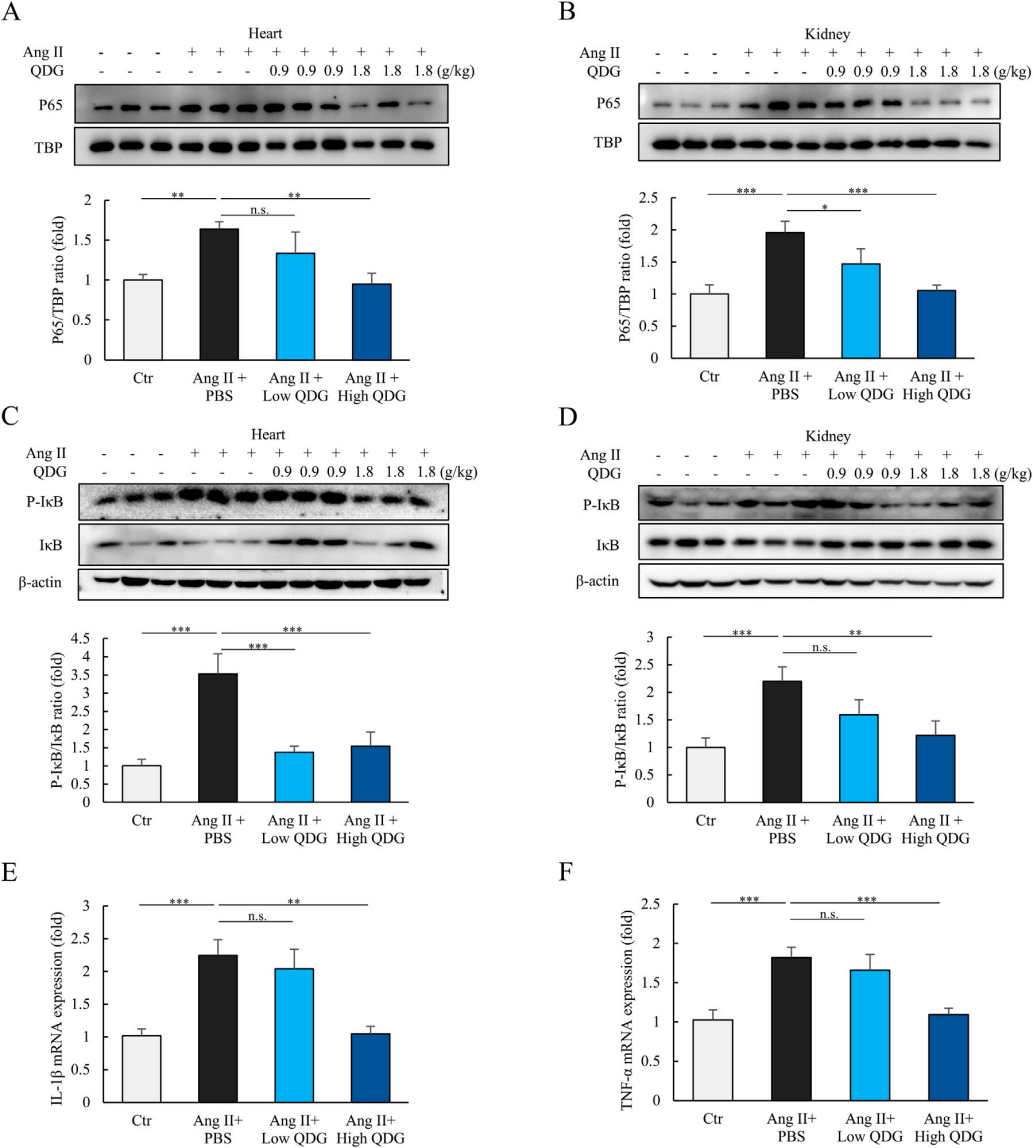
QDG inhibits Ang II/AT1R induced NF-κB signaling activation *in vivo*. **(A)** Representative immunoblots (upper) and quantification of nuclear P65 expression (lower) in the heart at 4 weeks post-Ang II infusion in mice administrated with PBS or QDG (0.9 g/kg/day, 1.8 g/kg/day). n = 6. n.s., no significance **P < 0.01 vs. Ang II + PBS. **(B)** Representative immunoblots (upper) and quantification of nuclear P65 expression (lower) in the kidneys of mice administrated with PBS or QDG at 4 weeks post-Ang II infusion. n = 6. *P < 0.05, ***P < 0.001 vs. Ang II + PBS. **(C)** Representative immunoblots (upper) and quantification (lower) of P-IκB and IκB expression in the heart of mice administrated with QDG (0.9 g/kg/day, 1.8 g/kg/day) or PBS for 3 days, and subsequent injection with Ang II (10 μg/kg, i.p.) for 10 min n = 6. ***P < 0.001 vs. Ang II + PBS. **(D)** Representative immunoblots (upper) and quantification (lower) of P-IκB and IκB expression in the kidneys of mice administrated with QDG or PBS for 3 days, and subsequent injection with Ang II (10 μg/kg, i.p.) for 10 min n = 6 n.s., no significance, **P < 0.01, ***P < 0.001 vs. Ang II + PBS. **(E,F)** *TNF-*α **(E)** and *IL-1β* **(F)** mRNA levels in the heart of mice treated as in **(A)**. n = 6. n.s., no significance, **P < 0.01, ***P < 0.001 vs. Ang II + PBS.

We further examined whether the ability of QDG in attenuating Ang II/AT1R activation also had an effect in regulating NF-κB signaling. Mice administered with chronic Ang II for 4 weeks led to the marked increase in the levels of nuclear P65 subunit of NF-κB in both the heart and kidneys, which were significantly prevented by daily QDG administration (Figures 6A,B). Furthermore, acute Ang II injection for 10 min also caused a rapid and significant increase in phosphorylated IκB (P-IκB) levels in both the heart and kidneys, which were effectively prevented in mice pre-treated with QDG (Figures 6C,D). Additionally, the mRNA levels of the downstream NF-κB targets, including pro-inflammatory cytokines *IL-1*β and *TNF-*α, were also markedly increased in the heart following chronic Ang II induction, and these were also prevented by QDG treatment (Figures 6E,F).

**FIGURE 7:**
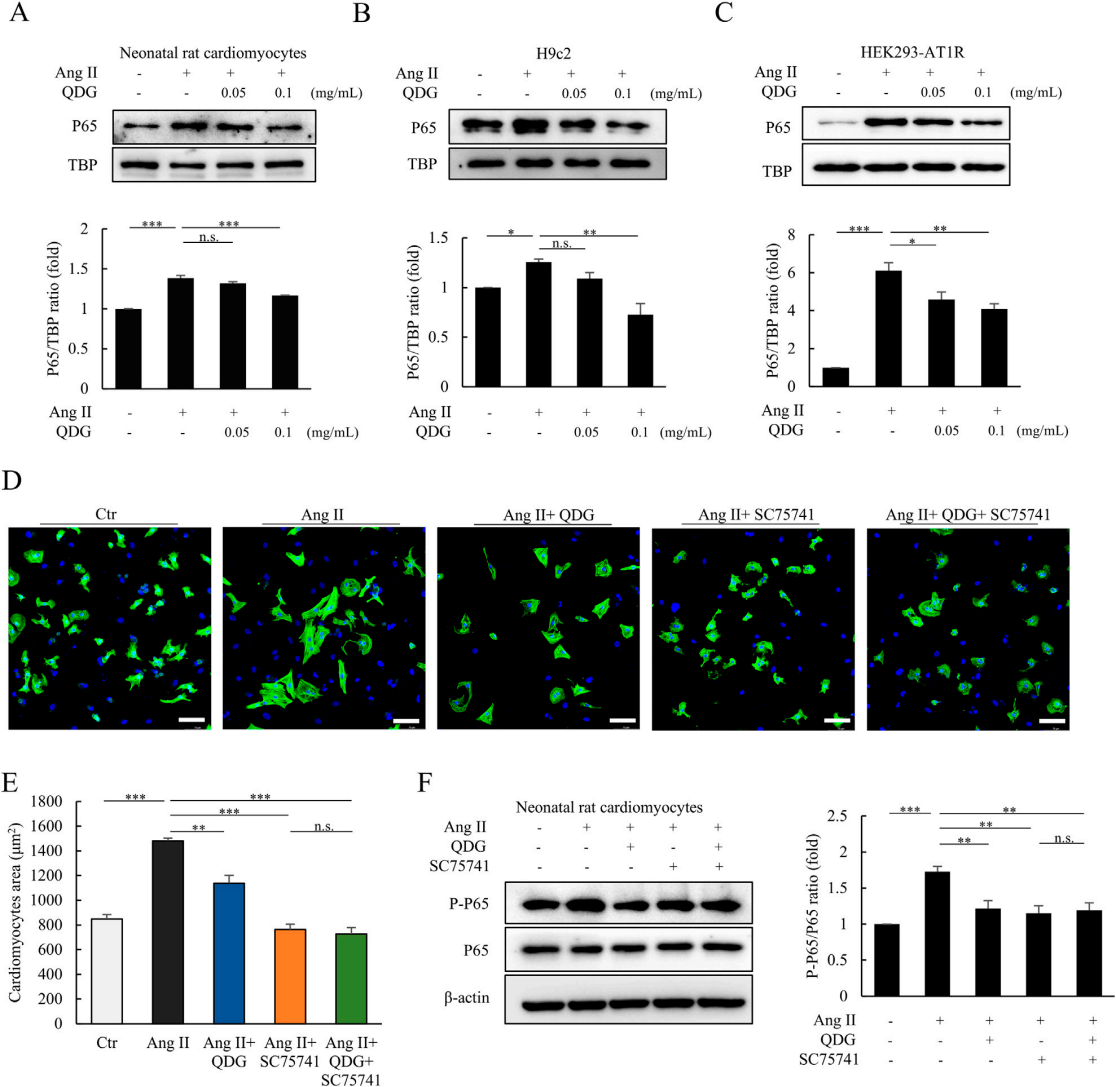
QDG inhibits Ang II/AT1R induced NF-κB signaling activation *in vitro*. **(A)** Representative immunoblots (upper) and quantification of nuclear P65 expression (lower) in NRCMs pretreated with QDG (0.05 mg/mL, 0.1 mg/mL) for 24 h and then treated with Ang II (1 μM) for 30 min n = 3. n.s., no significance, ***P < 0.001 vs. Ang II alone. **(B,C)** Representative immunoblots (upper) and quantification of nuclear P65 expression (lower) in H9c2 **(B)** and HEK293-ATIR **(C)** treated as in **(A)**. n = 3. *P < 0.05, **P < 0.01, ***P < 0.001 vs. Ang II alone. **(D)** Immunofluorescence images of NRCMs pretreated with QDG, SC75741 or both for 24 h and treated with PBS or Ang II for 24 h cTnT (green), DAPI (blue), scale bar 75 μm. **(E)** Quantification of cell area. NRCMs treated as in **(D)**. *P < 0.05, **P < 0.01, ***P < 0.001 vs. Ang II + PBS. n.s., no significance vs. Ang II + SC75741 **(F)** Representative immunoblots (left) and quantification (right) of P-P65 expression in NRCMs pretreated with QDG or SC75741 for 24 h and then treated with Ang II (1 μM) for 30 min n = 3. **P < 0.01, ***P < 0.001 vs. Ang II alone, n.s., no significance vs. Ang II + SC75741.

In addition, we explored the effects of QDG on the NF-κB signaling pathway *in vitro*. Ang II treatment induced rapid nuclear translocation of P65 within 30 min, which was prevented by QDG pre-treatment for 24 h in NRCMs (Figure 7A), H9c2 (Figure 7B), and HEK293-AT1R cells (Figure 7C). In order to verify whether QDG alleviates cardiac hypertrophy via regulating the NF-κB signaling pathway, we further used a selective NF-κB inhibitor, SC75741 in NRCMs following Ang II-induced cardiac hypertrophy. Immunofluorescence results showed that Ang II-induced enlargement of cardiomyocyte size was significantly prevented in cells pre-treated with SC75741 for 24 h (Figure 7D). Although the effect of QDG on Ang II-induced cardiomyocyte hypertrophy was less than SC75741, dual administration of QDG and SC75741 provided no additional benefits (Figures 7D,E), suggesting that QDG mainly exerts its effects via regulating NF-κB pathway. Furthermore, pre-treatment with QDG and/or SC75741 similarly attenuated acute Ang II-induced phosphorylated P65 (P-P65) levels (Figure 7F). In addition, we used TNF-α, an upstream modulator of NF-κB signaling to further support our findings that QDG acts via NF-κB inhibition. Interestingly, QDG treatment also significantly inhibited TNF-α-induced phosphorylation of both IκB and p65, although to a lower degree than the specific inhibitor SC75741 (Supplementary Figure S4A, S4B), suggesting that QDG mainly exerts its effects via inhibiting NF-κB signaling activation. Taken together, these results demonstrate that QDG prevents Ang II/AT1R-induced cardiac hypertrophic response via inhibiting NF-κB signaling activation.

## 4 Discussion

Ang II/AT1R signaling pathway plays a pivotal role in the development of cardiac hypertrophy and remodeling (Seo et al., 2020). Chronic Ang II stimulation causes compensatory cardiac hypertrophy that eventually progresses into decompensated pathophysiology (Bhullar and Dhalla, 2022). Our current study further revealed the decompensatory and adverse outcomes of chronic Ang II stimulation on cardiac hypertrophy, resulting in cardiac dysfunction and impairment. The finding that QDG, a formula used in Traditional Chinese Medicine, can robustly protect the heart from Ang II-induced cardiac hypertrophy, demonstrated its potential as a potential drug in Ang II-induced cardiac impairment and heart failure. Although previous studies also showed the protective ability of QDG in Ang II-induced cardiac hypertrophy (Cheng et al., 2021; Long et al., 2024), the underlying mechanisms of QDG’s beneficial effects have not been well elucidated. Our current study demonstrated for the first time that QDG’s cardioprotective effects were via robust inhibition of the NF-κB signaling pathway during Ang II-induced cardiac hypertrophy.

NF-κB signaling pathway activation is well-established to play a key role during cardiac hypertrophy (Freund et al., 2005). Previous studies showed that numerous drugs can inhibit Ang II-induced cardiac hypertrophy as well as the associated inflammation and oxidative stress via modulating the NF-κB pathway (Ren et al., 2021; Ma et al., 2023). Our current findings align with these studies, which demonstrated that QDG significantly reduces Ang II-induced nuclear translocation of nuclear P65 subunit of NF-κB, as well as prevent the elevations of pro-inflammatory cytokines *IL-1*β and *TNF-*α in the hearts of mice. Additionally, because AT1R is highly expressed in both the heart and kidneys (Lin et al., 2022), we further demonstrated that the effects of QDG in the kidneys mirrored those observed in the heart, which further supported our hypothesis that QDG inhibits Ang II/AT1R signal transduction via preventing NF-κB signaling activation. In addition, studies have showed that ERK signaling contributes to NF-κB signaling activation, whereby ERK promotes nuclear translocation of NF-κB by phosphorylating IκB (Roux and Blenis, 2004). Hence, our study findings that both Ang II-induced ERK1/2 phosphorylation and NF-κB activation are inhibited by QDG, provide further evidence of the mechanistic link between ERK and NF-κB signaling during Ang II/AT1R activation.

Another aspect of Ang II/AT1R signaling pathway activation is its well-characterized ability in constricting blood vessels and causing blood pressure elevation. Chronic hypertension is a major driver of pathologic left ventricular hypertrophy, often progressing to heart failure if left untreated (Gallo and Savoia, 2024). In this regard, QDG is a clinically established TCM drug in the treatment of hypertension. Although there are numerous clinical drugs used in the treatment of hypertension, recent studies suggest that these drugs do not actually prolong survival and may even cause potential long-term harmful effects (Ma et al., 2024). Therefore, discovering novel drugs that can limit the effects of Ang II-induced hypertension and associated cardiac hypertrophy and dysfunction is urgently needed. In this regard, QDG is a clinically established TCM drug in the treatment of hypertension. Hence, our study demonstrated QDG’s robust protective effects against Ang II-induced hypertension and its ability in preventing cardiac hypertrophy and cardiac dysfunction. There are a few key limitations to our study. Firstly, the high dose QDG group used 1.8 g/kg/day of extract based on the dose conversion formula for animals and humans. However, given the higher metabolic capacity in rodents, this dose may be excessive and prone to result in artefacts, although the low dose QDG group (0.9 g/kg/day) is maintained at a pharmacologically meaningful level whilst still providing notable efficacy. Secondly, there is a lack of detailed toxicity testing for the QDG extract used in our study. Even though there was no visible toxicity observed at both doses, further studies that comprehensively test for organ-specific toxicity are required.

Taken together, our findings elucidate the roles and mechanisms of QDG in Ang II-induced hypertension and cardiac hypertrophic response via preventing the activation of NF-κB signaling pathway. Our study thus underscores the potential clinical application of QDG as a novel drug in the treatment of pathological cardiac hypertrophy.

## Data Availability

The original contributions presented in the study are included in the article/Supplementary Material, further inquiries can be directed to the corresponding author.
