# Supplementary material for: Qingda granule prevents Ang II-induced cardiac hypertrophy via inhibiting NF-κB signaling pathway
Source: Front Pharmacol. 2025 Sep 16;16:1603316. doi: 10.3389/fphar.2025.1603316 (PMC12479541; doi:10.3389/fphar.2025.1603316)
Supplement: Supplementary file 1 [file DataSheet1.pdf]

Supplemental Figure. S1

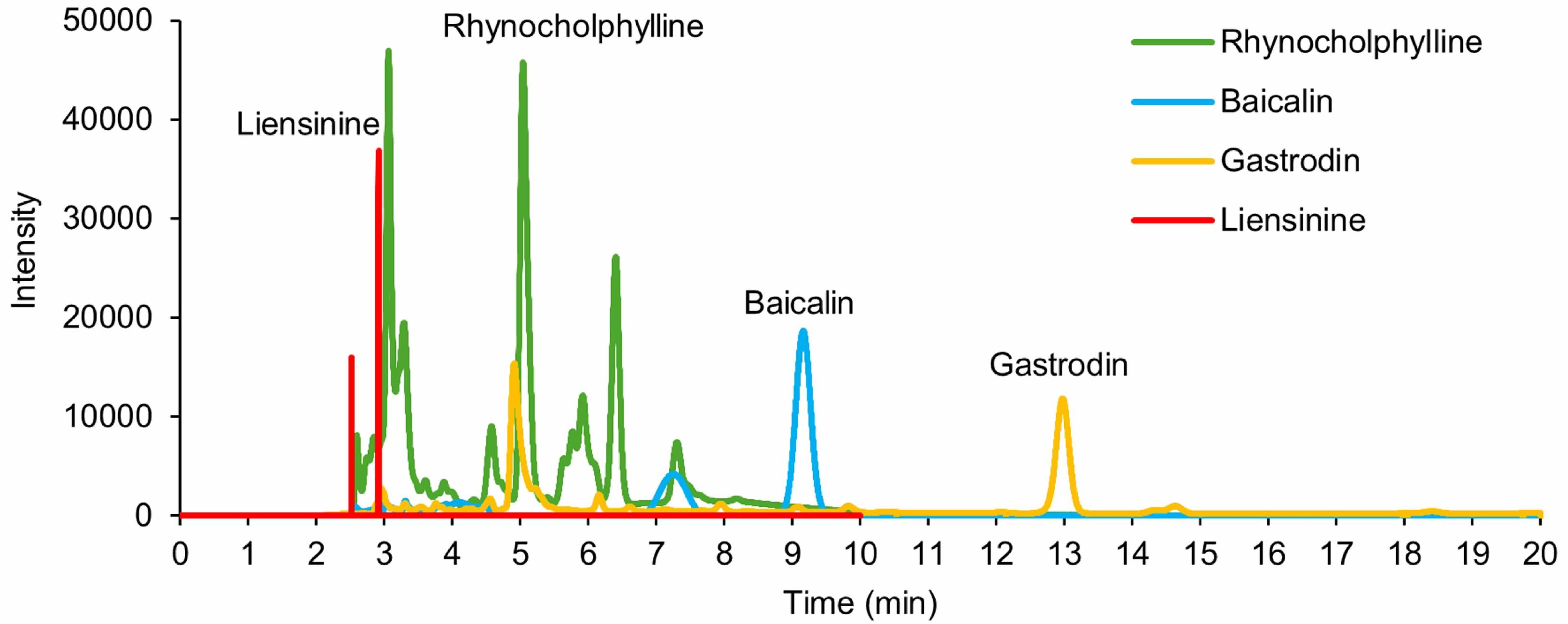

Supplemental Figure. S1 Chemical profiles of the top bioactive compounds contained within QDG formulation obtained via HPLC analysis, graphed according to their retention times and corresponding maximum intensity peaks, including liensinine, rhynocholphylline, baicalin, and gastrodin.

Supplemental Figure. S2

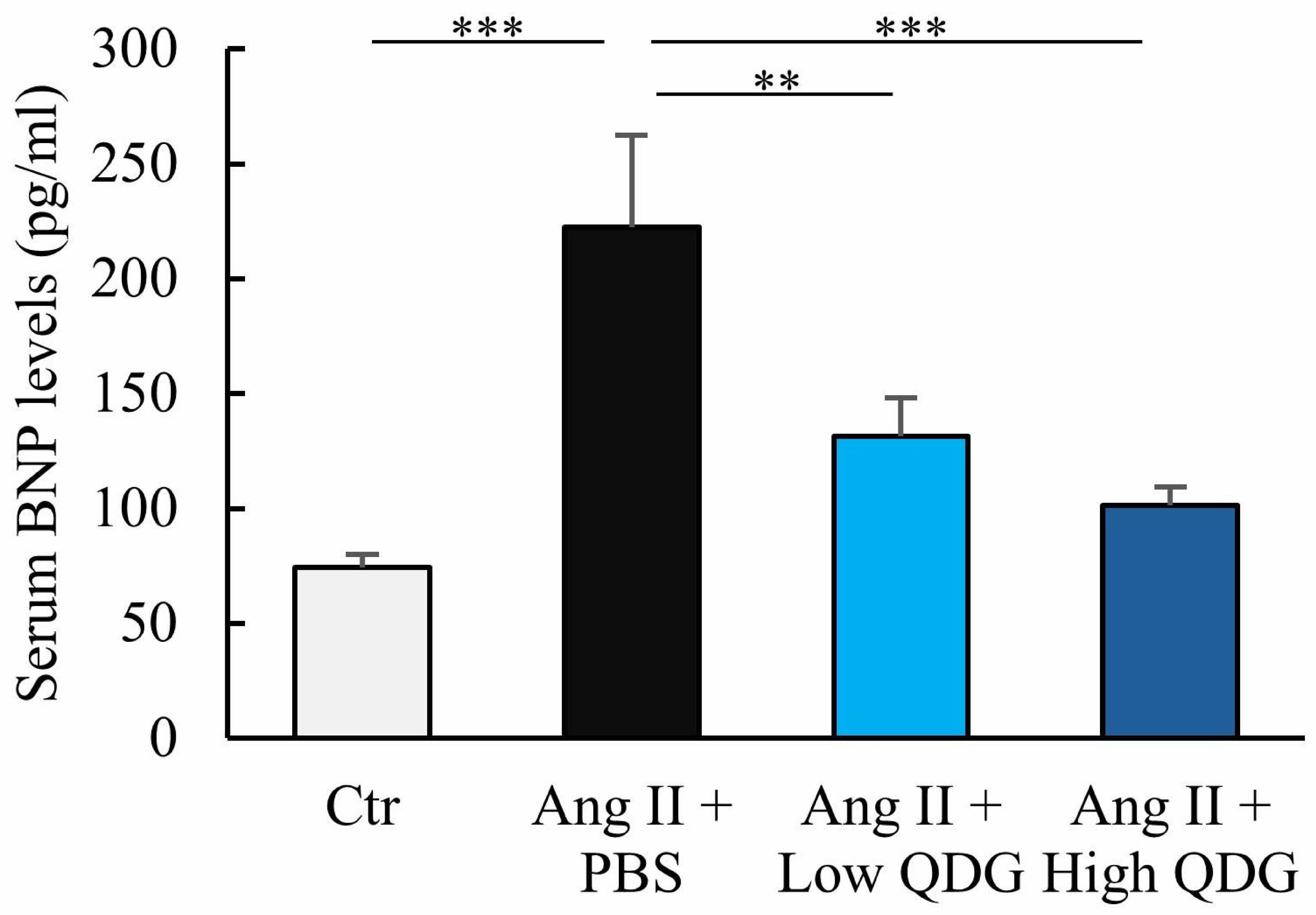

Supplemental Figure. S2 Levels of BNP in serum from mice administrated with PBS or QDG at 4 weeks post-Ang II infusion. n = 6. **$P < 0.01$, ***$P < 0.001$ vs Ang II + PBS.

Supplemental Figure. S3

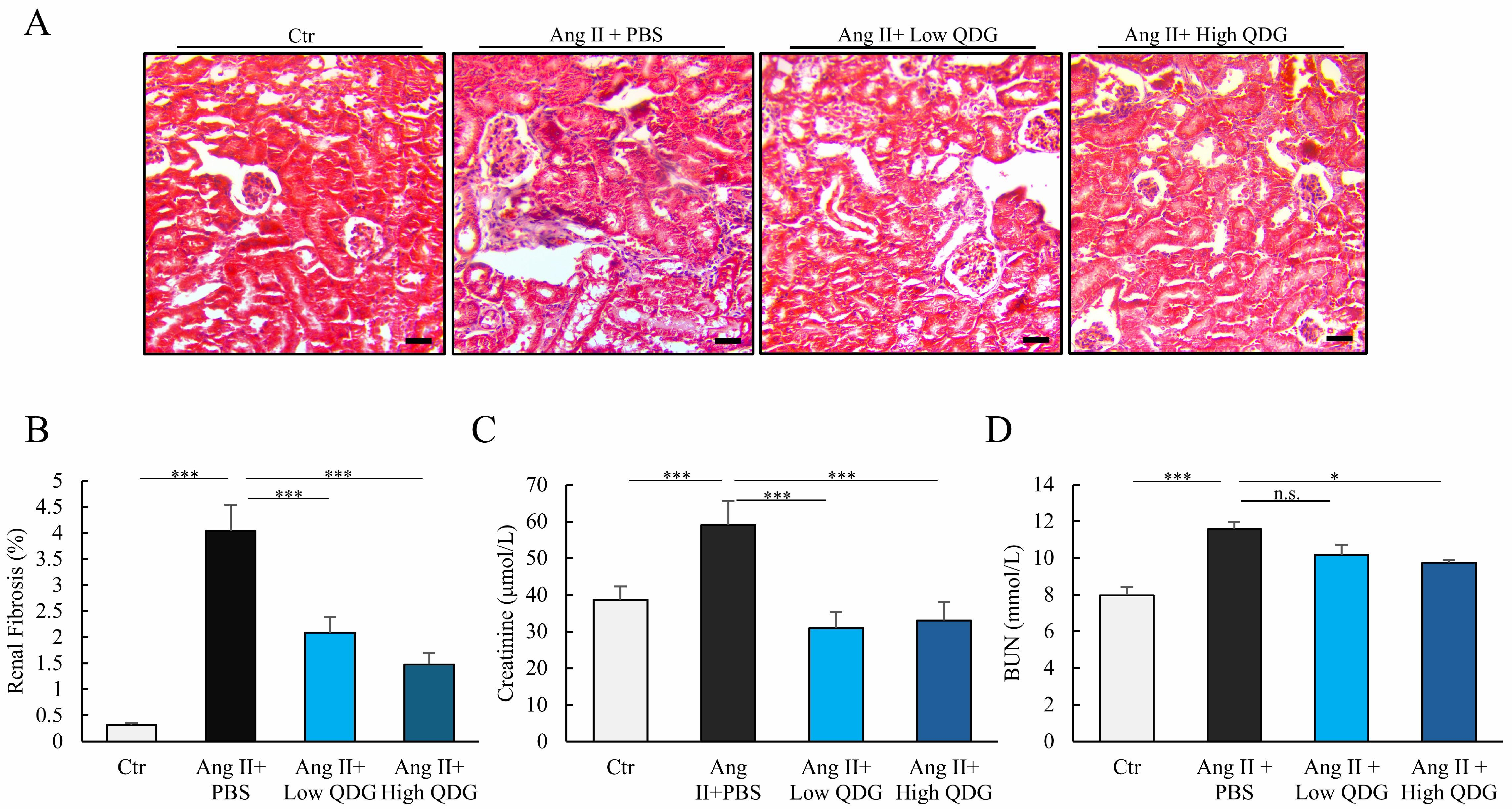

Supplemental Figure. S3 QDG prevents Ang II-induced renal fibrosis and dysfunction. (A) Masson's Trichrome Staining (scale bar, 20 μm) showing renal fibrosis in mice administrated with PBS or QDG at 4 weeks post-Ang II infusion. (B) Quantification of renal fibrosis in mice treated as in (A). n = 8. ***P < 0.001 vs Ang II + PBS. (C-D) Creatinine (C), BUN (D) levels in serum from mice administrated with PBS or QDG at 4 weeks post-Ang II infusion. n = 6. n.s. >0.05, *P < 0.05, ***P < 0.001 vs Ang II + PBS.

Supplemental Figure. S4

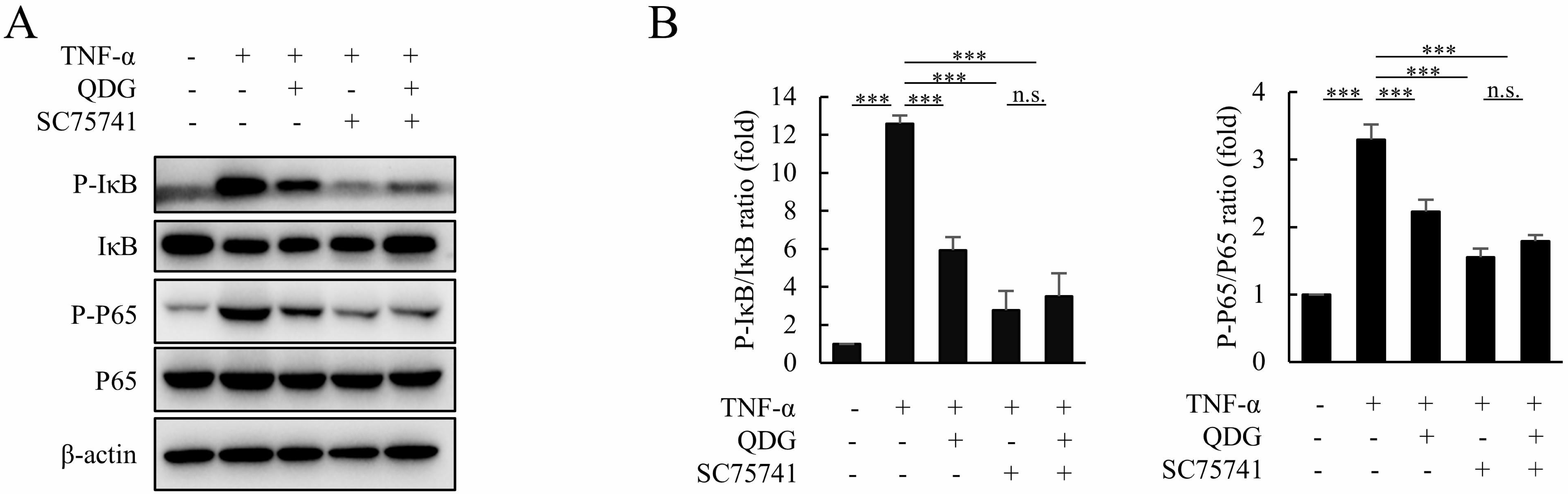

Supplemental Figure. S4 QDG inhibits TNF-α induced NF-κB signaling activation *in vitro*. (A) Representative immunoblots of phosphorylated P-IκB and P-P65 expression in NRCMs pretreated with QDG (0.1 mg/ml), SC75741 (10 μM) or both for 24 hours and then treated with TNF-α (10 μg/ml) for 4 hours. (B) Quantification of P-IκB (left) and P-P65 (right) expression in NRCMs treated as in (A). n = 3. ***P < 0.001 vs TNF-α + PBS. n.s. > 0.05 vs TNF-α + SC75741.
